# The impact of hyperglycemia on urinary albumin excretion in recent onset diabetes mellitus type II

**DOI:** 10.1186/s12882-020-01774-0

**Published:** 2020-04-06

**Authors:** Barbara Nikolaidou, Eugenia Gkaliagkousi, Panagiota Anyfanti, Eleni Gavriilaki, Antonios Lazaridis, Areti Triantafyllou, Ioanna Zografou, Stella Douma

**Affiliations:** 1 3rd Department of Internal Medicine, Papageorgiou Hospital, Aristotle University of Thessaloniki, Ring Road Nea Efkarpia, 56429 Thessaloniki, Greece; 2 2nd Propedeutic Department of Internal Medicine, Hippokration Hospital, Aristotle University of Thessaloniki, Thessaloniki, Greece

**Keywords:** Urinary albumin excretion, Arterial stiffness, Hypertension, Diabetes, Hyperglycemia

## Abstract

**Background:**

Increased urinary albumin excretion (UAE) in diabetes is a sensitive marker of microvascular injury and a reliable predictor of cardiovascular outcomes. Hypertension-induced hemodynamic pressure load, diabetes-related metabolic processes and large artery stiffening have all been implicated in the development of microalbuminuria. We investigated whether hyperglycemia per se, or rather increased blood pressure (BP) and macrovascular dysfunction, is a stronger predictor of UAE at the earliest stages of diabetes.

**Methods:**

Consecutive newly diagnosed patients with diabetes type 2, who were normoglycemic within a year’s time prior to diagnosis, were enrolled. UAE was estimated in 24-h urine samples. Both office and 24-h ambulatory BP was recorded. Arterial stiffness was evaluated by measurement of carotid-femoral pulse wave velocity (PWV) with applanation tonometry.

**Results:**

Among 71 newly diagnosed patients with median diabetes duration of just 1 month, 15.5% presented microalbuminuria. UAE did not differ between hypertensive and normotensive diabetics; however, newly diagnosed patients for both hypertension and diabetes exhibited significantly higher levels of UAE, compared to diabetic patients with long-standing hypertension. UAE strongly and significantly correlated with office systolic BP, HbA1c, PWV and estimated glomerular filtration rate. However, in the multivariate analysis adjusting for these factors, only HbA1c was independently associated with UAE (beta = 0.278, *p* = 0.049).

**Conclusions:**

Hyperglycemic state emerges as a powerful predictor of increased UAE even at the earliest stages of diabetes. The relative contribution of hypertension and macrovascular dysfunction to the development of microalbuminuria seems to be obscured by hyperglycemia, even in patients whose diabetes onset does not exceed a few months’ time.

## Background

Microalbuminuria, typically defined as urinary albumin excretion (UAE) of 30–300 mg in a 24-h urine sample, corresponds to a subclinical microvascular derangement in the glomerular filtration barrier that precedes overt diabetic nephropathy [1]. It has been well-established that increased UAE, even below the threshold values usually considered for microalbuminuria, constitutes an early marker of microvascular damage and a potent predictor of cardiovascular morbidity and mortality [2].

Apart from diabetes, hypertension represents a major risk factor for increased UAE. When both diseases coincide in the same individual, which is typically the case for the vast majority of patients with diabetes mellitus type II (DMII), the risk of developing microalbuminuria is substantially aggravated [3]. Another contributor that has been proposed for the development of microalbuminouria, is arterial stiffness and the interplay between micro- and macro-vascular dysfunction. In line with this, several studies have demonstrated an association between microalbuminuria and arterial stiffness in DMII and other populations [4–6]. However, the majority of studies have included patients with long-standing or unclear duration of the disease. Importantly, DMII-related complications, including microalbuminuria and arterial stiffness, increase with disease duration [7, 8]. Of note, most patients with DMII already suffer from hypertension at the time of diagnosis, which substantially increases the risk for microalbuminuria.

Altogether, it remains unclear as to which extent hyperglycemia, increased blood pressure (BP) and macrovascular dysfunction, contribute to microalbuminuria in the very early stages of DMII, long before the establishment of the disease-related cardiovascular complications. Hence, the aim of our study was to investigate the association of increased UAE with high BP, hyperglycemia and arterial stiffness in a meticulously selected population of newly-diagnosed patients with DMII.

## Methods

We enrolled consecutive patients recently diagnosed with Diabetes Mellitus type II (less than 6 months from diagnosis). We excluded patients with cardiovascular disease or stroke, chronic kidney disease [glomerular filtration rate (GFR) ≤45 ml/min/m2], inflammatory diseases, and pregnancy, as well as patients that had or were receiving antidiabetic treatment were excluded, with the only exception of Metformin. DMII diagnosis was documented two impaired glucose tests in separate days [fasting blood glucose (FBG) ≥ 126 mg/dl and/or 2-h post-load glucose ≥200 mg/dl in oral glucose tolerance test (OGTT, 75 g)] [9, 10]. We also investigated medical records of all participants as well as FBG or OGTT values to confirm that they were normal for up to 1 year earlier in order to establish the recent diagnosis of DMII. All participants were Caucasian and gave written informed consent. The study was conducted in accordance with the Helsinki declaration and approved by the local Institutional Ethics Committee.

A thorough medical history was recorded with emphasis on comorbidities and medication use and physical examination was performed. Body mass index (BMI) was calculated in kg/m^2^. Office systolic/diastolic BP (SBP/DBP) was measured with a validated oscillometric device (Microlife Exact BP, Microlife AG, Widnau, Switzerland) in the sitting position according to the guidelines [11]. Ambulatory BP monitoring (ABPM) was performed in the non-dominant arm, on a working day with a validated oscillometric device Spacelabs 90217A (Spacelabs Medical, Issequah, WA, USA). BP readings were obtained every 20 min during daytime (awake period: 06:00–22:00 h) and every 30 min during night-time (asleep period: 22:00–06:00 h). Subjects were instructed to take note of retiring to bed in a diary. The actual sleep and wake intervals of each patient were redefined according to their statement on the day of device removal. Measurements were used for the analysis only if > 70% of the recordings were valid. Hypertension was defined according to the guidelines as ambulatory daytime BP ≥135/85 mmHg and/or current antihypertensive medication use [11].

After completion of the procedures, as analyzed below, blood samples were collected to estimate glycemic state, fasting lipids and renal function. GFR was estimated using the Cockcroft-Gault formula [12].

### - Urinary albumin excretion and microalbuminuria

UAE was measured with the immuno-turbidimetric method in 24-h urine samples, which is considered as the gold standard for the diagnosis of microalbuminuria [13]. UAE of 30–300 mg/24 h was defined as microalbuminuria. Participants were advised against strenuous exercise and any change in their usual dietary habits.

### - Assessment of arterial stiffness

Carotid-femoral pulse wave velocity (PWV) was assessed as the gold-standard measure of arterial stiffness using applanation tonometry with the Sphygmocor device (AtCor Medical, Sydney, Australia). Sequential recordings of the arterial wave were obtained in the carotid and femoral artery and wave transit time was calculated with a simultaneously recorded electrocardiogram. PWV was determined by the distance difference between sternal notch to carotid site and sternal notch to femoral site b divided by time (PWV = Δd/Δt). Participants were advised to abstain from caffeine, smoking and intense physical activity for at least 3 h before the procedure, which was performed in the supine position after a 15-min resting period.

### - Statistical analysis

Analysis was performed using the Statistical Package for Social Sciences (SPSS), version 22. Results were expressed as frequencies for qualitative variables and as mean ± standard deviation (m ± SD) or median (1st quartile–3rd quartile) for continuous variables, according to whether they were normally distributed or not. Comparison of frequencies was performed with the Pearson chi-square test for qualitative variables, while mean values were compared with Student t or Mann–Whitney test for continuous variables. Correlation coefficients were calculated with the parametric Pearson or the non-parametric Spearman rank tests, according to the normality of their distribution. The logarithmic mean of the parameter was entered where needed to transform a non-normal distribution to a normal distribution. Multiple linear regression analysis was used to identify independent associations with UAE. A probability value of *p* ≤ 0.05 was considered statistically significant.

#### Patient and public involvement

There was no patient or public involvement in this research study.

## Results

A total of 71 newly diagnosed patients with DMII, 41 males and 30 females with a mean age of 56.6 ± 10.4 years, were included. Baseline characteristics of the study population are depicted in Table 1. Median disease duration from the time of diagnosis was only 1 month. Microalbuminuria affected 15.5% of our population, which corresponded to median UAE values of 7.5 (2.9–17.3) mg. As expected, the majority of our patients (62.0%) presented concomitant hypertension. In particular, 27 patients (38.0%) had a history of long-standing hypertension with median duration of 8 (4–12) years, while 17 (23.9%) were simultaneously diagnosed with hypertension and DMII. Treatment with metformin had been initiated in 47.9%, while 22.5% were receiving renin-angiotensin-aldosterone system (RAAS) inhibitors.

**Table 1:** Baseline characteristics of the study population

Variable	All patients (***n*** = 71)	Hypertensive patients with diabetes (***n*** = 44)	Normotensive patients with diabetes (***n*** = 27)	***P*** value
Age (years)	56.6 ± 10.4	57.1 ± 11.3	55.7 ± 8.9	0.584
Male sex (%)	57.7	59.8	59.3	0.840
Duration of diabetes (months)	1 (0–5)	0 (0–3.5)	1 (0–6)	0.199
Fasting glucose (mg/dl)	120 (104–137)	121 (100.5–133.5)	112 (105.5–155)	0.691
HbA1c (%)	6.7 (6.3–7.5)	6.7 (6.3–7.4)	6.7 (6.3–7.7)	0.803
Metformin (%)	47.9	45.5	51.9	0.600
BMI (kg/m^2^)	30.3 ± 4.2	31.1 (28.5–33.8)	29.5 ± 3.9	0.049
Duration of hypertension (years)	8 (4–12)	8 (4–12)	ΝΑ	ΝΑ
Office SBP (mmHg)	137.6 ± 20.5	142.5 ± 21.1	129.0 ± 16.2	0.008
Office DBP (mmHg)	84.0 ± 11.9	87.0 ± 12.7	78.8 ± 8.1	0.002
Daytime SBP (mmHg)	129.2 ± 16.1	134.5 ± 16.7	120.0 ± 9.8	< 0.001
Daytime DBP (mmHg)	79.1 ± 11.5	82.0 ± 12.8	74.1 ± 6.3	0.001
Nighttime SBP (mmHg)	120.2 ± 16.7	125.3 ± 16.9	110.2 ± 11.1	< 0.001
Nighttime DBP (mmHg)	71.3 ± 10.8	73.9 ± 11.3	66.3 ± 8.1	0.007
UAE (mg/24 h)	7.5 (2.9–17.3)	9.1 (3.7–17.8)	5.1 (2.5–8.7)	0.147
PWV (m/s)	8.9 ± 1.8	8.8 (7.3–10.2)	8.5 ± 1.3	0.087
Total cholesterol (mg/dl)	190.9 ± 38.4	191.4 ± 36.3	190.0 ± 42.5	0.880
LDL cholesterol (mg/dl)	121.2 ± 33.8	121.9 ± 29.0	120.0 ± 41.2	0.826
HDL cholesterol (mg/dl)	41.3 ± 11.1	41.0 ± 10.9	41.8 ± 11.8	0.749
Triglycerides (mg/dl)	122 (102–174)	134.5 (103.5–187)	113.0 (102–161)	0.208
Urea (mg/dl)	31.2 ± 6.9	31.2 ± 7.1	31.1 ± 6.8	0.927
Creatinine (mg/dl)	0.88 ± 0.16	0.86 ± 0.15	0.92 ± 0.18	0.114
GFR (mL/min/1.73 m^2^)	111.6 ± 34.8	116.7 ± 38.0	103.4 ± 27.5	0.126
Uric acid (mg/dl)	5.4 ± 1.7	5.5 ± 1.5	5.2 ± 1.99	0.458
Antihypertensive medication (%)			NA	NA
RAAS inhibitors (%)	22.5	36.4	NA	NA
b-blockers (%)	2.8	4.5	NA	NA
Calcium channel blockers (%)	8.5	13.6	NA	NA

*BMI* Body Mass Index, *SBP* Systolic Blood Pressure, *DBP* Diastolic Blood Pressure, *UAE* Urinary Albumin Excretion, *PWV* Pulse Wave Velocity, *LDL* Low-Density Lipoprotein, *HDL* High-Density Lipoprotein, *GFR* Glomerular Filtration Rate, *RAAS* Renin-angiotensin-aldosterone system, *ΝΑ* Not Applicable

Results are demonstrated as Mean ± SD / Median (IQ range)

Results of univariate correlation analysis of UAE with demographic, metabolic and blood pressure parameters are depicted in Table 2. In this very meticulously selected group of newly diagnosed diabetic patients, UAE correlated with all the known factors, which contribute to its progression in patients with long standing DMII, including HbA1c, FBG, office SBP and DBP, as well as day- and nighttime SBP (Table 2). Although levels of UAE did not significantly differ between diabetics with or without hypertension [9.1 (3.65–17.8) vs 5.1 (2.5–8.7), *p* = 0.111], patients who were simultaneously diagnosed with both hypertension and DMII exhibited significantly higher levels of UAE, compared to diabetics who presented long-standing hypertension [13.3 (9.0–58.0) vs 7.0 (25–7.5) mg, *p* = 0.007].

**Table 2:** Results of univariate correlation analysis of urinary albumin excretion with blood pressure, demographic, metabolic and biochemical parameters

Variable	r	*P* value
Age (years)	− 0.040	0.748
Duration of diabetes (months)	−0.169	0.332
Fasting glucose (mg/dl)	0.250	0.040
HbA1c (%)	0.330	0.006
BMI (kg/m^2^)	0.176	0.153
Office SBP (mmHg)	0.357	0.003
Office DBP (mmHg)	0.350	0.004
Daytime SBP (mmHg)	0.285	0.021
Daytime DBP (mmHg)	0.235	0.055
Nighttime SBP (mmHg)	0.270	0.034
Nighttime DBP (mmHg)	0.229	0.073
PWV (m/s)	0.287	0.019
Total cholesterol (mg/dl)	−0.065	0.602
LDL cholesterol (mg/dl)	− 0.130	0.293
HDL cholesterol (mg/dl)	−0.098	0.429
Triglycerides (mg/dl)	0.088	0.481
GFR (mL/min/1.73 m^2^)	0.306	0.013
Uric acid (mg/dl)	0.086	0.497

*BMI* Body Mass Index, *SBP* Systolic Blood Pressure, *DBP* Diastolic Blood Pressure, *PWV* Pulse Wave Velocity, *LDL* Low-Density Lipoprotein, *HDL* High-Density Lipoprotein, *GFR* Glomerular Filtration Rate

Moreover, a positive association was observed between UAE and macrovascular dysfunction, represented by PWV (Table 2). When patients were further classified according to the presence of microalbuminuria, PWV was significantly higher in the microalbuminuria group, compared to patients with normal UAE (10.2 ± 1.9 vs 8.7 ± 1.8 m/s, *p* = 0.013). Finally, increasing UAE was associated with renal function, reflected in GFR (Table 2). Non-significant associations were observed between UAE and other cardiovascular risk factors (age, gender, BMI, lipids, uric acid).

Multiple regression analysis for UAE adjusting for glycemia, BP levels, renal function and macrovascular impairment revealed that HbA1c was the only factor that was independently associated with UAE (beta = 0.278, *p* = 0.049), in our population of newly diagnosed patients with DMII (Table 3).

**Table 3:** Linear regression model for urinary albumin excretion (UAE) in newly-diagnosed patients with diabetes

Variable	Unstandardized Coefficients		Standardized Coefficients	95% Confidence Intervals for B		***P*** value
	B	SD	Beta	Lower Bound	Upper Bound	
Age (years)	−0.447	0.757	−0.115	−1.962	1.068	0.557
Gender	−6.938	11.601	−0.084	−30.159	16.283	0.552
Office SBP (mmHg)	0.090	0.305	0.043	−0.520	0.700	0.770
BMI (kg/m^2^)	29.362	112.409	0.043	− 195.649	254.374	0.795
HbA1c (%)	8.629	4.295	0.278	0.032	17.226	0.049
PWV (m/s)	2.040	3.603	0.092	−5.171	9.251	0.573
GFR (mL/min/1.73 m^2^)	−0.130	0.257	−0.110	−0.644	0.383	0.614

^a^Dependent variable: UAE; Statistic F = 1.033, *p* = 0.418, R^2^ = 0.111, adjusted R^2^ = 0.004

*SBP* Systolic Blood Pressure, *BMI* Body Mass Index, *PWV* Pulse Wave Velocity, *GFR* Glomerular Filtration Rate

## Discussion

The most important finding of the present study is that even at the earliest stages of DMII, hyperglycemia emerges as a powerful predictor of increased UAE and seems to negate the well-known detrimental effects of hypertension, compromised renal function as well as macrovascular dysfunction regarding microalbuminuria development and progression. To our knowledge, this is the first study to address the influence of the above factors on microalbuminuria in a carefully selected population of patients with such a short duration of DMII (a median of just 1 month from the time of diagnosis). These results imply that neither the concomitant diagnosis nor the long-standing history of hypertension can overcome the detrimental effect of hyperglycemia even at the very early stages of DMII. Importantly, only patients who were normoglycemic within a year’s time prior to the diagnosis were included, in order to verify the recent onset of DMII. Even though the rate of DMII-related microvascular complications increases with the duration of disease [7, 8] the present study highlights that renal endothelial dysfunction, reflected in increased UAE, rapidly progresses at the time of diagnosis and is mainly triggered by hyperglycemia.

The observed associations of UAE with BP and arterial stiffness merit further attention. Both were strongly and positively associated with UAE in the univariate analysis. However, the strength of these associations was attenuated and they were subsequently rendered non-significant after adjustment for HbA1c (Table 3). Considering the adverse effects of hypertension on UAE and especially the fact that BP in our sample was not optimal, our results point towards metabolic, rather than hemodynamic processes, as the major mechanism underlying the development of microalbuminuria at the earliest stages of DMII. Hence, the relative contribution of hypertension seems to be obscured by hyperglycemia in patients with such recent onset of DMII.

Likewise, the association between arterial stiffness and UAE no longer remained significant after adjustment for HbA1c. Increased arterial stiffness has been proposed as an additional mechanism triggering microalbuminuria in patients with DMII, through increased intrarenal pulsatile stress and enhanced hemodynamic load in the renal tubules and glomeruli induced by large artery stiffening. This concept has been reinforced by several cross-sectional studies demonstrating an independent association between microalbuminuria and arterial stiffness in patients with DMII [14–16]. However, the duration of DMII in these studies was either uncertain or relatively long, and just a single study enrolled patients whose disease duration did not exceed 12 months [17]. Concordantly, DMII duration exceeded 10 years in a prospective study showing that arterial stiffness was associated with incident albuminuria [4]. It is thus likely that in long-standing disease, the arterial wall properties within the micro- and macrovasculature have undergone alterations that are mutually linked and reinforced. By contrast, based on our study findings, it is reasonable to presume that in early-stage DMII, the interaction between UAE and arterial stiffness is mediated by hyperglycemia, which appears as the common denominator of microvascular damage.

Mechanisms responsible for microalbuminuria in hyperglycemic state include endothelial dysfunction and chronic inflammation triggered by oxidative stress, inflammatory cytokines and growth factors [18]. Glycation of structural proteins in DMII, such as collagen and elastin, with the formation of advanced glycation end products (AGEs), affects arterial compliance and has been proposed as an additional mechanism of microalbuminuria [19]. Since hyperglycemia was identified as the only independent predictor of UAE in our sample, our study suggests that these mechanisms seem to be activated within a few months’ time following DMII onset. However, insulin resistance that has been likewise implicated in the genesis of albuminuria [18], might have been present for a considerable period of time, before the clinical diagnosis of DMII.

The strengths of our study include our meticulously selected population of patients with very short duration of DMII, which is extremely difficult to find in the available literature, and the use of the gold-standard methods for the assessment of UAE and arterial stiffness. Presence of hypertension was not based on conventional office BP measurements, but was verified with 24-h ABPM recordings. Our study is limited by its cross-sectional design and the relatively small sample size. Consequently no conclusions can be deduced from the present study regarding potential associations between UAE and medication. Hence, our study might be considered rather as a pilot study, and further studies with a larger number of participants need to confirm our results. However, the difficulty of identifying patients with such short duration of DMII, who are free from significant comorbidities, needs to be acknowledged.

## Conclusions

In conclusion, our study identified increased HbA1c as the major mediator of renal microvascular impairment at the earliest stages of DMII. In patients with disease onset that does not exceed a few months’ time, hyperglycemia seems to obscure the observed associations of UAE with BP, renal function and arterial stiffness. These results stress out the need for early implementation of effective interventions targeting at this powerful modifiable predictor of microalbuminuria.

## Data Availability

The dataset analyzed during the present study are available from the corresponding author on reasonable request.

## Abbreviations

ABPM: Ambulatory Blood Pressure Monitoring
AGEs: Advanced Glycation End Products
BMI: Body Mass Index
BP: Blood Pressure
DBP: Diastolic Blood Pressure
DMII: Diabetes Mellitus type II
FBG: Fasting Blood Glucose
GFR: Glomerular Filtration Rate
HbA1c: Glycated Haemoglobin
OGTT: Oral Glucose Tolerance Test
PWV: Pulse Wave Velocity
RAAS: Renin-Angiotensin-Aldosterone System
SBP: Systolic Blood Pressure
SD: Standard Deviation
SPSS: Statistical Package for Social Sciences
UAE: Urinary Albumin Excretion

## References

[1] Parving HH (1996). Initiation and progression of diabetic nephropathy. N Engl J Med.

[2] Karalliedde J, Viberti G (2004). Microalbuminuria and cardiovascular risk. Am J Hypertens.

[3] Hallan H, Romundstad S, Kvenild K, Holmen J (2003). Microalbuminuria in diabetic and hypertensive patients and the general population--consequences of various diagnostic criteria--the Nord-Trondelag health study (HUNT). Scand J Urol Nephrol.

[4] Bouchi R, Babazono T, Mugishima M, Yoshida N, Nyumura I, Toya K (2011). Arterial stiffness is associated with incident albuminuria and decreased glomerular filtration rate in type 2 diabetic patients. Diabetes Care.

[5] Kim BJ, Lee HA, Kim NH, Kim MW, Kim BS, Kang JH (2011). The association of albuminuria, arterial stiffness, and blood pressure status in nondiabetic, nonhypertensive individuals. J Hypertens.

[6] Gkaliagkousi E, Anyfanti P, Triantafyllou A, Gavriilaki E, Nikolaidou B, Lazaridis A (2018). Aldosterone as a mediator of microvascular and macrovascular damage in a population of normotensive to early-stage hypertensive individuals. J Am Soc Hypertens.

[7] Huang E, Laiteerapong N, Liu J, John P, Moffet H, Karter A (2014). Rates of complications and mortality in older patients with diabetes mellitus the diabetes and aging study. JAMA Intern Med.

[8] Agnoletti D, Mansour A, Zhang Y, Protogerou A, Ouerdane S, Blacher J (2017). Clinical interaction between diabetes duration and aortic stiffness in type 2 diabetes mellitus. J Hum Hypertens.

[9] Report and Consultation (WHO) WHO (2011). Abbreviated report of a WHO consultation. Use of Glycated Haemoglobin (HbA1c) in the Diagnosis of Diabetes Mellitus.

[10] Consultation. Definition and diagnosis of diabetes and intermediate hyperglycemia: World Health Organization (WHO); 2006. https://www.who.int/diabetes/publications/Definition%20and%20diagnosis%20of%20diabetes_new.pdf.

[11] Mancia G, Fagard R, Narkiewicz K, Redón J, Zanchetti A, Böhm M (2013). 2013 ESH/ESC guidelines for the management of arterial hypertension the task force for the management of arterial hypertension of the European Society of Hypertension (ESH) and of the European Society of Cardiology (ESC). J Hypertens.

[12] Cockcroft D, Gault M (1976). Prediction of creatinine clearance from serum creatinine. Nephron..

[13] De Jong PE, Curhan GC (2006). Screening, monitoring, and treatment of albuminuria: public health perspetives. J Am Soc Nephrol.

[14] Smith A, Karalliedde J, De Angelis L, Goldsmith D, Viberti G (2005). Aortic pulse wave velocity and albuminuria in patients with type 2 diabetes. J Am Soc Nephrol [Internet].

[15] Ishimura E, Taniwaki H, Tsuchida T, Obatake N, Emoto M, Shoji T, et al. Urinary albumin excretion associated with arterial wall stiffness rather than thickness in type 2 diabetic patients. J Nephrol, Available from: http://www.ncbi.nlm.nih.gov/pubmed/17514625. 20(2):204–11. 17514625

[16] Sjöblom P, Nystrom FH, Länne T, Engvall J, Östgren CJ (2014). Microalbuminuria, but not reduced eGFR, is associated with cardiovascular subclinical organ damage in type 2 diabetes. Diabetes Metab.

[17] Shin DI, Seung K-B, Yoon HE, Hwang B-H, Seo SM, Shin SJ (2013). Microalbuminuria is independently associated with arterial stiffness and vascular inflammation but not with carotid intima-media thickness in patients with newly diagnosed type 2 diabetes or essential hypertension. J Korean Med Sci.

[18] Satchell SC, Tooke JE (2008). What is the mechanism of microalbuminuria in diabetes: a role for the glomerular endothelium?. Diabetologia..

[19] Miranda-Díaz AG, Pazarín-Villaseñor L, Yanowsky-Escatell FG, Andrade-Sierra J (2016). Oxidative stress in diabetic nephropathy with early chronic kidney disease. J Diabetes Res.

